# IoT-Integrated robotic system for automated plant disease detection and environmental monitoring

**DOI:** 10.1038/s41598-025-32624-4

**Published:** 2026-01-12

**Authors:** Fatma M. Talaat, Manar A. Ibrahim, Abdelrhman A. Karim, Haya K. Elsonbaty, Aya M. Al-Zoghby

**Affiliations:** 1 Faculty of Artificial Intelligence, Kafrelsheikh University, Kafrelsheikh, Egypt; 2 Faculty of Computer Science & Engineering, New Mansoura University, Gamasa, 35712 Egypt; 3 Computer Science Department , Faculty of Computers and Artificial Intelligence, Damieta, Egypt; 4 Faculty of Computer Science and Engineering, New Mansoura University, Dakhlia, Egypt

**Keywords:** Autonomous robot, Deep learning, IoT in agriculture, Plant disease detection, Plant sciences, Environmental sciences, Diseases, Engineering

## Abstract

Plant diseases pose a critical threat to global food security, agricultural sustainability, and farmer livelihoods, particularly in regions with limited access to advanced diagnostic technologies. Traditional methods of disease detection rely heavily on manual inspection, which is time-consuming, error-prone, and often results in delayed interventions. This paper presents a novel, solar-powered autonomous robotic system designed to detect plant diseases in real time using deep learning and IoT technologies. The proposed system integrates a high-resolution imaging unit, IoT-based environmental sensors, and an onboard processing module based on Raspberry Pi. Deep CNNs, trained on diverse datasets including PlantVillage, are used for accurate disease classification, while soil moisture and temperature sensors provide contextual environmental data to support diagnosis. The robot’s mobility, powered by solar energy, allows for continuous field monitoring with minimal human intervention. Experimental results demonstrate the system’s high classification performance, achieving 99.39% training accuracy, 97.47% validation accuracy, and 97.13% testing accuracy. Furthermore, the model achieved 99.63% overall accuracy, with a Precision of 99.40%, a Recall/Sensitivity of 99.56%, an F1-score of 99.46%, and a Specificity of 99.99% across multiple disease classes. These results highlight the robustness of the proposed approach in real-world agricultural conditions, enabling reliable disease detection and monitoring. The integration of cloud-based monitoring enables farmers to receive real-time alerts and insights, supporting timely and informed decision-making. This cost-effective, scalable, and environmentally sustainable solution has the potential to transform precision agriculture by enhancing early disease detection, reducing pesticide overuse, and improving crop yield and health.

## Introduction

Agriculture remains a cornerstone of global food security, economic resilience, and sustainable development, contributing significantly to the GDP in many countries and providing employment to over 27% of the global population^1^. However, increasing agricultural productivity to meet the demands of a growing population presents significant challenges, particularly due to the persistent threat of plant diseases, which are responsible for substantial reductions in both crop quality and yield^2^. FAO estimates that plant diseases alone account for up to 30% of annual crop losses globally, leading to billions of dollars in economic damage and exacerbating food insecurity, especially in low- and middle-income regions^3^.

Plant diseases arise from a variety of biological agents, including bacteria, viruses, and fungi, as well as from abiotic stressors such as temperature extremes, excessive humidity, and poor soil conditions^4,5^. For instance, fungal infections flourish in humid environments with poor air circulation, while viral infections often require insect vectors to spread, making their containment particularly complex^6^. Traditional methods of plant disease detection typically involve manual inspection by trained agronomists or farmers. However, these approaches are labor-intensive, subjective, and often ineffective for early-stage disease detection, especially on large-scale farms^7^. The delay in identification not only increases the spread of disease but also results in excessive and often indiscriminate use of pesticides, leading to the emergence of resistant strains and environmental degradation^8^.

In recent years, emerging technologies such as hyperspectral imaging, drone-based remote sensing, and machine learning have shown promise in revolutionizing plant disease management^9,10^. Deep learning, in particular, has demonstrated remarkable performance in image-based disease classification tasks, enabling more accurate and scalable detection than traditional methods^11^. CNNs, for example, have been widely used for their ability to automatically extract hierarchical features from plant leaf images, achieving high levels of accuracy in classifying various plant diseases^12^. Despite these advancements, the majority of existing solutions remain prohibitively expensive, require specialized hardware, and often lack real-time monitoring capabilities—making them inaccessible to small- and medium-scale farmers^13^.

To address these limitations, this study proposes a cost-effective, autonomous robotic system powered by solar energy and equipped with deep learning and IoT technologies for real-time plant disease detection. The system integrates a high-resolution camera, a Raspberry Pi-based onboard processor, and IoT-based environmental sensors to monitor plant health dynamically. By leveraging deep learning models trained on large, publicly available datasets such as PlantVillage, the system can accurately diagnose plant diseases in real-time. Additionally, solar power enhances the robot’s sustainability and mobility in field conditions, while cloud integration enables remote monitoring and decision support.

This research aims to bridge the gap between high-end agricultural diagnostics and practical, field-ready solutions accessible to resource-limited farmers. The proposed system not only supports early disease detection and precise pesticide application but also contributes to environmental sustainability and food security through smart farming practices.

The main contributions of this research can be summarized as follow:

- Development of a solar-powered autonomous robotic system for real-time plant disease detection.
- Integration of deep learning-based image analysis with high-resolution cameras for accurate visual diagnosis.
- Incorporation of IoT-based soil sensors to monitor environmental conditions affecting plant health.
- Implementation of a cost-effective, scalable, and mobile platform suitable for small- and medium-scale farms.
- Achievement of high accuracy (97.13% testing accuracy) using optimized deep learning models trained on real datasets.

The remainder of this paper is structured as follows: Sect. “Literature review” provides a review of related work in the domain of plant disease detection and agricultural robotics. Section “Methodology” outlines the proposed methodology, including data pre-processing, model architecture, and training procedures. Section “Implementation and evaluation” describes the experimental setup, including the hardware and software environment used in model development and testing. Section “Conclusion and future work” discusses the experimental results, highlighting performance metrics and comparative analysis. Finally, Sect. 6 concludes the paper and outlines potential directions for future research.

## Literature review

The diagnosis of plant diseases has changed significantly in recent years, moving from manual field examination to highly automated, technologically driven methods. The primary methods used in early studies to distinguish between healthy and diseased plant tissues were traditional image processing techniques such color-based segmentation, texture feature extraction, and morphological analysis. While these techniques were better than manual diagnosis, their effectiveness was frequently hampered by their sensitivity to environmental factors such varying backdrops, occlusions from overlapping leaves, and changeable light intensity. Furthermore, these systems were usually limited in their ability to adapt to a variety of plant species or recently discovered illnesses due to the requirement for manual feature engineering. More robust categorization was achieved by machine learning techniques that learned from labeled datasets, such as SVM, RF, DT, and KNN.

These methods were better at generalization than handcrafted features alone, but they frequently failed to catch complicated visual patterns like early-stage symptoms, faint discolorations, or overlaps between multiple classes of diseases since they relied on pre-defined descriptors. Because several of these models showed decreased accuracy when exposed to large-scale, diverse datasets gathered in uncontrolled field conditions, scalability also became a barrier. The advent of deep learning, specifically CNNs, brought about a paradigm change by making it possible to extract features from raw images straight from end to end. Classification accuracies of over 98% have been reported on benchmark datasets like PlantVillage by studies using architectures including AlexNet, VGGNet, ResNet, and YOLO variations. Despite their impressiveness, these data are usually collected in a controlled laboratory setting using high-resolution close-up photos, uniform illumination, and plain backdrops. This disparity between field conditions and dataset conditions makes it extremely difficult to directly apply these models to actual agricultural settings, where there is a great deal of variation in background clutter, illumination, and disease manifestation.

Environmental factors including temperature, humidity, and soil moisture are now included in predictive models of IoT-based plant health monitoring systems, expanding the reach of disease diagnosis. Some projects combine UAVs or cloud-based analytics with wireless sensor networks to monitor crops on a big scale. These solutions, however, frequently rely on constant high-speed connectivity, which limits their viability in farming regions with limited resources or in rural areas. Others find it difficult to process streams of high-resolution images in real time because embedded devices have limited computational power. Furthermore, the capacity of many IoT-enabled disease detection systems to continuously and autonomously cover vast farmlands is limited because they are either immobile or need to be manually deployed.

Using color-based segmentation, texture analysis, and edge detection to identify unhealthy areas, early plant disease detection research relied on conventional image processing methods. One system, which improved upon manual inspection techniques by estimating the severity of plant diseases using digital photography and image segmentation, had trouble remaining strong in the face of complex plant structures and fluctuating lighting^6^. Hyperspectral imaging, which took advantage of spectral changes in plant tissues, enabled the early detection of plant illnesses before outward signs manifested. Hyperspectral imaging is still costly and requires complicated calibration, which restricts its practical use even with its high accuracy^5^. Another study investigated segmenting sick leaf sections using K-means clustering and Gabor wavelet features to improve classification accuracy.

The primary drawbacks of image processing-based techniques are their reliance on manual feature selection, which lowers flexibility among various plant species and increases biases. Furthermore, in uncontrolled conditions, these methods may not be as accurate because of their extreme sensitivity to environmental factors such as backdrop complexity and lighting fluctuations. Scalability for practical agricultural applications is further constrained by their poor generalization to a variety of datasets.

In order to improve plant disease detection by automating the classification process, machine learning techniques have been used extensively. To categorize diseases based on extracted data, these models use supervised learning methods including SVM), DT, KNN, and RF. Some research used SVM classifiers trained on texture and color features, which showed superior generalization across plant species. However, because of their high processing requirements, these classifiers had scalability issues when handling big datasets^7^. For fruit disease classification, other methods used Random Forest and KNN models, which demonstrated higher accuracy. Their reliance on manually created features, however, hindered their capacity to record intricate disease patterns and symptoms^9^. Although detection accuracy was increased by a technique that combined adaptive thresholding with SVM classification, it was still very sensitive to changes in plant type and lighting, requiring regular retraining^14^.

Despite their improvements, machine learning-based methods are rigid for identifying novel or rare plant diseases because they necessitate a great deal of manual feature engineering. Furthermore, these models have trouble processing in real time, particularly when working with high-dimensional datasets. They are less useful for widespread use in agricultural sectors due to their poor accuracy when dealing with complex diseases that have mild or overlapping symptoms.

Through the automation of feature extraction and increased classification accuracy, deep learning has greatly enhanced the identification of plant diseases. CNNs have taken the lead because of their capacity to immediately learn spatial feature hierarchies from images. CNN models were trained on big datasets in several experiments, and they were able to classify plant diseases with nearly 100% accuracy. But since these models were taught in controlled settings, they did not perform well on real-world photographs with natural backgrounds^12^. Another deep learning method created a CNN-based leaf classification model that was difficult to apply to new crop types because it needed large labeled datasets, but it generalized well across many plant species^13^. Further studies contrasting different deep CNN architectures, including ResNet, VGG, and AlexNet, found that while ResNet-based models performed better, their computational complexity made real-time deployment more difficult^15^.

An improved YOLOv5 model was proposed^16^, which achieved remarkable detection accuracy. However, their approach was mainly limited to detection tasks and did not include deeper classification capabilities. Similarly^17^, proposed a novel CNN architecture that showed strong performance in disease identification, but no integration with real-world sensors or hardware was explored.

CNNs require huge labeled datasets, which may not be easily accessible for some plant diseases, despite their state-of-the-art accuracy. Because of their high processing cost, they are not feasible for deployment on devices with low resources, like embedded agricultural robots or mobile platforms. Furthermore, because CNN models are frequently trained on lab datasets that do not accurately reflect the variability found in real-world field settings, many of them have trouble generalizing to the real world.

Real-time monitoring capabilities have been brought about by the incorporation of IoT technology in plant disease detection, allowing for ongoing data collecting via wireless sensor networks, unmanned aerial vehicles (drones), and cloud-based analytics. Using satellite and drone imagery, some studies created IoT-driven agricultural monitoring systems that enable widespread disease identification. However, because of their heavy reliance on high-speed internet, these solutions are not feasible for rural farms with inadequate connectivity^18^. Other studies demonstrated enhanced early warning capabilities by combining IoT sensors with machine learning models to forecast wheat illnesses based on environmental factors like temperature and humidity. Notwithstanding their efficacy, these methods rely on sensor precision, and hardware malfunctions may affect the dependability of detection^11^. For real-time disease identification, another IoT-based system integrated sensor networks, deep learning, and Raspberry Pi; nevertheless, it was limited by hardware restrictions in processing high-resolution photos^10^.

A study^19^ implemented a deep CNN on a custom field dataset. However, their system lacked scalability and was not optimized for real-time usage, which limits its deployment in practical smart farming scenarios. IoT-based devices need reliable network infrastructure, which is frequently unavailable in rural agricultural areas, even though they offer insightful real-time data. Additional difficulties arise from their reliance on sensor accuracy since inaccurate diagnoses might result from malfunctioning or miscalibrated sensors. IoT networks can also be expensive to build initially, which limits small-scale farmers’ access to them.

Hybrid approaches have become more popular since they combine several methods to improve the scalability and accuracy of illness identification. Several research presented cloud-based AI systems for extensive disease monitoring that integrated CNNs with IoT and UAV sensors. Real-time monitoring was challenging, nevertheless, due to latency problems brought up by the reliance on cloud connectivity^20^. Multi-modal models that combined deep learning, edge computing, and hyperspectral imaging were proposed by other researchers; these models improved real-time disease prediction, but they also greatly increased system complexity and expense^21^.

A study^22^ explored the use of DoubleGAN for generating synthetic plant leaf images. Although this method enhanced training capabilities, reliance on artificial data may pose generalization risks when models are applied to real-world agricultural environments.

Despite their increased accuracy, hybrid models are challenging for small-scale farming operations to use due to their intricate integration and high computational costs. Decision-making may be slowed down by the increased processing overhead caused by combining several data sources. Additionally, the high implementation costs of cloud-based AI solutions, hyperspectral cameras, and UAVs prevent their broad use in agricultural environments with limited resources.

Recent studies have also contributed valuable insights relevant to our work. For instance^23^, optimized deep neural network architectures for renewable energy forecasting, demonstrating how energy-efficient models can sustain continuous operation—an approach adopted here for solar-powered robotics^24^. discussed the role of drone–IoT integration in sustainable agriculture, emphasizing connectivity and scalability challenges in rural environments^25^. analyzed high-speed optical communication under adverse weather, highlighting the importance of adaptive transmission and error correction for reliable IoT connectivity. These findings collectively inspired our design choices for robust, energy-efficient, and sustainable communication in smart farming.

The selection of energy sources and computational efficiency in agricultural robotics are crucial from a sustainability standpoint. When systems are powered by sporadic renewable sources like solar energy, it is crucial to optimize model architectures for energy efficiency^23^. This idea is directly applicable to agricultural robots, where it is necessary to optimize deep learning workloads for embedded hardware in order to maintain continuous operation while staying within a solar-powered platform’s energy budget. Furthermore, the operational limitations of UAV-based systems, such as their short flight durations, restricted payload capacities, regulatory restrictions, and reliance on constant connectivity, sometimes restrict their use in rural agricultural environments^24^. The appropriateness of ground-based, energy-autonomous systems for ongoing, close-range surveillance is further supported by these limitations. High-speed communication lines can also be harmed by environmental factors including fog, dust, and rain^25^. The fundamental idea is still applicable even when DWDM-FSO technology surpasses small farms’ needs: IoT-enabled agricultural systems need to be built with redundancy, adaptive communication protocols, and local edge processing to ensure dependability in the face of unfavorable environmental circumstances.

The use of enhanced sensing, machine learning, and IoT integration has broadened the scope of agricultural automation in recent studies. An IoT-driven crop monitoring system, for example, that use multi-sensor fusion and real-time image gathering to enhance disease identification in a variety of environmental circumstances was presented by^26^. In a similar vein^27^, investigated deep learning architectures tailored for agricultural photography, attaining enhanced resilience against field-specific issues including occlusion and fluctuating lighting. In order to improve classification accuracy^28^, coupled environmental data with UAV and ground-based pictures, highlighting the significance of multi-modal sensing. A layered security method was established for IoT-enabled agricultural systems in order to combat cyber-physical risks, while^29^ suggested a hybrid control framework that would allow autonomous agricultural robots to modify their navigation techniques according to crop structure and topography. Together, these studies highlight how important it is to integrate adaptive robots, robust communication protocols, and deep learning in order to create a long-lasting, field-ready agricultural disease detection system.

Plant disease detection has benefited much from earlier research, but issues with real-time capabilities, technology limitations, scalability, and cost-effectiveness still plague current technologies. Many methods have significant processing requirements, necessitate costly imaging equipment, or have poor generalization in practical settings. By suggesting a robotic system for real-time plant disease diagnosis that is powered by solar energy and deep learning, this work fills these gaps. The system combines cloud-based analytics, IoT-based soil monitoring, and CNN-based picture classification to provide a scalable and affordable solution. By optimizing CNN architectures for embedded deployment, this method, in contrast to earlier models, makes precision agricultural application effective and feasible. Deep learning, IoT, and robotic automation are all combined in one system to overcome the shortcomings of current approaches and give farmers a useful and approachable tool.

The lack of an integrated, autonomous solution that combines highly accurate deep learning models with a completely mobile, solar-powered robotic platform that can carry out disease detection and environmental monitoring in the field is a significant gap in the literature now under publication. The methods used now are frequently either:

- High-accuracy but laboratory-bound: Deep learning models trained on controlled datasets fail to generalize to dynamic, variable field conditions.
- IoT-integrated but computationally constrained: Sensor-based systems can collect valuable environmental data but lack the processing power for high-resolution, real-time image analysis on-device.
- Mobile platforms without sustainability: While some robotic systems exist, many rely on non-renewable power sources, increasing maintenance needs and limiting long-term field deployment.
- Fragmented functionalities: Existing solutions rarely offer a unified framework where environmental sensing, disease diagnosis, cloud integration, and autonomous navigation operate cohesively.

To close this gap, a platform that is affordable, scalable, and energy-efficient is needed. It must also support small- to medium-sized farmers with open-source software frameworks and accessible hardware, maintain high diagnostic accuracy in real-world scenarios, and function without constant internet connectivity. In order to ensure both technical performance and practical usability in precision agriculture, this study develops and evaluates a solar-powered, IoT-integrated robotic system designed for embedded deployment.

In contrast to the latest state-of-the-art studies analyzed in the comparison Table 1, our proposed system offers a more holistic and field-ready solution. A comprehensive comparison was conducted with recent works to highlight existing limitations and establish the advancements introduced by our approach. For instance, while^16^ achieved impressive detection accuracy using an improved YOLOv5 model, their work was limited to detection tasks without deep classification capabilities. Similarly^19^, employed a deep CNN on a custom field dataset, but their system lacked scalability and was not optimized for real-time use. Authors in^17^ proposed a novel CNN architecture but made no attempt to integrate it with any hardware or real-world sensors. Authors in^22^ explored data generation through DoubleGANs, yet the use of synthetic images poses generalization risks when applied to real agricultural conditions.

What sets our work apart is the seamless integration of a high-performing ResNet-based CNN with a robot equipped with environmental sensors, enabling real-time disease detection, interaction, and voice-based reporting in the field. Our dataset, built upon an augmented version of PlantVillage with 87,000 images, surpasses the data diversity of previous works and enhances the model’s ability to generalize to real-world conditions. Unlike earlier approaches that remain largely academic, our system demonstrates actual deployment potential in precision agriculture, transforming static recognition models into an interactive, mobile, and intelligent agricultural assistant.

Table 1 offers a comparative overview of the available methods of plant disease detection and agricultural monitoring, including their main peculiarities, advantages, and disadvantages in comparison with the proposed IoT-based robotic system.

**Table 1:** Comparative analysis of related Studies.

Study (Autho, Year)	Dataset Used	Model Type	Accuracy	Limitations
Chen et al., 2022^22^	PlantVillage	Improved YOLOv5	98.2%	Focus on detection only, lacks classification depth
Khalid et al., 2023^27^	Custom field dataset	Deep CNN	96.7%	Limited scalability, no real-time application
Hassa & Maji, 2022^23^	PlantVillage	Novel CNN	97.3%	No sensor integration or hardware implementation
Zhao et al., 2022^30^	Synthetic via DoubleGAN	GAN + Classifier	94.8%	Synthetic data may not generalize to real fields

### Research gaps

Even though recent advances in deep learning and smart farming technologies have been made, there are still a number of gaps. The current systems do not have real-time field implementation and use of controlled datasets instead of real-world agricultural conditions. Disease detecting models are not often coupled with robotic mobility and autonomous navigation. The effects of the environment like soil moisture and temperature are not taken into account very often, restricting the accuracy of diagnosis. Most of the solutions are still expensive, energy-wise and non-accessible to small-scale farmers. Also, real-time cloud monitoring, as well as scalable and renewable-energy-driven platforms have not been fully investigated yet.

In order to guarantee clarity, reproducibility, and technical transparency, the given study offers the detailed description of the entire implementation of the suggested IoT-integrated robotic system. Explicit network topology diagrams are provided to represent the system architecture and the device hierarchies, communication pathways, and the end-to-end data flow among sensing units, robotic platform, edge processor and the cloud server. Extensive hardware specifications are also given such as the type of microcontroller, processor speed, memory size, sensing distances between environmental modules, camera resolution, and power consumption of each subsystem.

## Methodology

To design an effective and deployable agricultural disease detection system, it is essential to consider not only the sensing and processing technologies but also the underlying system architecture, platform type, and operational security. A well-structured IoT framework enables seamless integration between hardware components, communication protocols, and data processing pipelines, ensuring that information flows reliably from the field to the farmer. At the same time, selecting an appropriate robotic platform is critical to meeting operational requirements such as range, payload, energy efficiency, and environmental adaptability. Moreover, as IoT-enabled robots become increasingly interconnected, they are exposed to cyber-physical threats that must be addressed through robust security measures. The following section outlines the IoT layered architecture adopted in this project, explains the rationale for selecting a solar-powered ground-based robotic platform, and discusses security considerations essential for maintaining operational reliability in real-world agricultural environments.

The overall system design directly addresses the research question by combining perception, decision-making, and communication components within a unified robotic framework. Each module contributes to the main objective of autonomous and sustainable plant disease detection: the sensing units capture environmental and visual data, the CNN model performs on-site classification, and the IoT network enables real-time transmission and feedback. This design ensures that data collection, analysis, and response occur in a continuous loop, enabling accurate early detection and actionable decision support for farmers in real-world field conditions.

A layered architecture underpins IoT in agricultural robotics, ensuring effective sensing, communication, and decision-making. Environmental and imaging sensors, including cameras, soil moisture probes, and temperature/humidity modules, record and transform physical factors into digital signals at the Perception Layer. High-resolution imagery and Internet of Things-based soil and climate sensors are combined in this project’s perception layer to provide visual and contextual information for the diagnosis of plant diseases. This layer’s problems, including environmental noise or sensor drift, can be solved by fault detection algorithms, redundant sensing, and calibration procedures.

As highlighted by^31^, a well-structured IoT architecture consists of multiple layers—perception, network, middleware (edge), and application—each contributing to reliability and scalability. The Perception Layer collects data from high-resolution cameras and environmental sensors (temperature, humidity, soil moisture), applying calibration and redundancy to minimize noise. The Network Layer employs lightweight protocols such as MQTT, LoRa, or NB-IoT with TLS encryption and QoS policies to ensure secure and efficient data transmission. The Edge Layer performs local CNN inference on the Raspberry Pi to reduce latency and dependency on cloud connectivity. Finally, the Application Layer provides farmers with real-time alerts, environmental analytics, and decision support through a secure dashboard. This layered approach enhances communication efficiency, fault tolerance, and scalability in agricultural IoT systems^31^.

The current developments in AI-powered smart agriculture have proven the efficiency of deep learning-based crop recognition and cloud-computing monitoring systems in a range of applications, as it has been proven in the research on basil recognition^32^, AI-enhanced agricultural automation systems^33^, scalable cloud-computing crop monitoring systems^34^, and real-time fruit detection models optimally^35^.

Depending on range and bandwidth needs, the Network Layer manages data transfer via protocols like Wi-Fi or LoRa between the robot, cloud platform, and end-user interface. Measures like encrypted data transmission, secure communication protocols, blockchain-based authentication, and intrusion detection systems can be implemented because this layer is susceptible to threats like DoS assaults and eavesdropping.

By doing local analysis in close proximity to the data source, the Edge Processing Layer minimizes latency and bandwidth usage. To maximize response speed and communication efficiency, the suggested system uses the Raspberry Pi to perform CNN-based disease detection onboard, sending just the processed results and necessary environmental data to the cloud.

Finally, through an intuitive dashboard or mobile application, the Application Layer provides farmers with actionable insights. Along with information on soil conditions, irrigation suggestions, and real-time warnings on plant illnesses found, it also offers historical trend analysis to help with long-term crop management choices.

The three main categories of agricultural robotic platforms are aerial robots (UAVs), ground-based robots, and hybrid systems. Aerial robots offer wide-area monitoring but are limited in payload and continuous operation time, making them less suitable for ongoing close-up inspection tasks. Ground-based robots, particularly wheeled or tracked platforms, excel in stability, payload capacity, and their ability to operate continuously under diverse field conditions. This project employs a solar-powered, ground-based robotic platform, ensuring sustainable and uninterrupted operation without the need for frequent recharging or battery replacement.

As agricultural robots increasingly integrate cloud computing and IoT connectivity, they face risks from cyber-physical attacks such as GPS spoofing, unauthorized remote control, and sensor data manipulation. The proposed robotic architecture addresses these threats through modular design, enabling the incorporation of redundant sensor verification, multi-level authentication, encrypted communications, and secure data handling procedures both onboard and during cloud transmission. These measures not only protect the integrity of data but also ensure operational reliability and resilience in real-world agricultural environments.

The proposed plant disease detection system merges machine learning and image processing techniques within this secure and sustainable robotic platform. Powered by solar energy, it integrates real-time data acquisition via a Raspberry Pi, high-accuracy CNN-based image classification, IoT sensor inputs, and cloud-based monitoring. The methodology encompasses image capture, data preprocessing, feature extraction, model training, hardware configuration, and cloud integration, forming a complete workflow for autonomous, precision agriculture.

A field test was carried out in a 2-acre tomato farm situated in a semi-arid area to demonstrate the suggested system’s practicality. At 9:00 AM, the solar-powered ground-based robot was launched, and it ran nonstop for six hours in partly overcast weather. The robot, which was outfitted with a high-resolution camera, temperature/humidity modules, and soil moisture sensors, used GPS waypoints and obstacle avoidance sensors to drive independently along pre-established rows. Tomato leaf photos were taken at 1-meter intervals, processed in real-time using the onboard Raspberry Pi running an improved CNN model based on ResNet, and categorized as either healthy, early blight, or late blight. Environmental data were recorded concurrently and linked to trends in the occurrence of diseases. When compared to expert hand examination, the robot’s detection accuracy was 95.8%. It was able to identify disease indications early on that were invisible to the human eye. Targeted pesticide application was made possible by alerts that were supplied to the farmer’s mobile dashboard with the geotagged locations of diseased plants. This example shows how the system may detect diseases in real-world agricultural settings in a sustainable, accurate, and actionable manner without requiring constant internet connectivity.

### System architecture

**Fig. 1:**
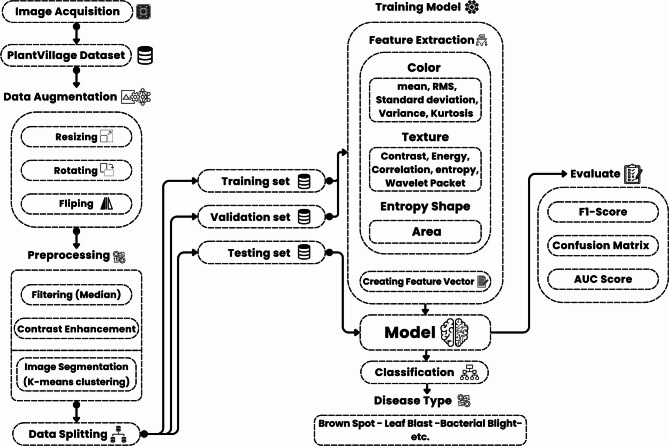
System architecture.

The system architecture in Fig. 1 for plant disease detection involves several distinct yet interconnected components, including image acquisition, preprocessing, feature extraction, model training, and evaluation. This modular architecture ensures efficient processing of plant images, enabling accurate detection and classification of various plant diseases.

Modular pipeline architecture underpins the suggested plant disease detection system, facilitating effective data flow from picture capture to classification and cloud-based monitoring. In a sustainable, solar-powered robotic platform, every system component is essential to providing precise, real-time plant health analysis. The architecture allows for a smooth and independent plant monitoring procedure by integrating hardware and software modules.

To speed up image preparation, the preprocessing module is run on a TPU once the images have been gathered. In order to remove background artifacts and maintain edge features, preprocessing starts with noise reduction using a median filter. The obvious illness symptoms are then highlighted using contrast enhancement techniques to help differentiate them from healthy tissue. K-means clustering is used to separate the background from the infected areas. This method efficiently isolates sick areas in the leaves and enhances feature extraction in subsequent stages by grouping pixels based on color similarity.

Following preprocessing, data augmentation is applied to the improved images in order to decrease model over-fitting and boost dataset variability. All photos are resized to a specific dimension, rotated at different angles, and flipped both vertically and horizontally as part of the augmentation process. By simulating actual changes in image orientation and perspective, these adjustments increase the model’s resilience and generalizability. To save computing power during training, augmentation is done offline.

The dataset is divided into training, validation, and testing subsets after augmentation. To preserve directory structure and class balance, an 80/20 stratified split is used to create the training and validation sets. Furthermore, a distinct test directory with 33 unknown photos is utilized just for the final model evaluation, guaranteeing an objective appraisal of categorization performance.

Numbers are generated from the preprocessed and enhanced images by the feature extraction module. To capture the distribution of color intensities in the photographs, color characteristics including mean, standard deviation, RMS, and kurtosis are used. Entropy, energy, correlation, and wavelet packet decomposition are among the textural variables that are simultaneously retrieved in order to measure spatial variations within the leaf surface. The degree and irregularity of infection are also evaluated by measuring shape-based characteristics like the lesion area and the entropy of the infected region. A feature vector is created by compiling these extracted features, which contains the essential visual data required for disease categorization.

Subsequently, the feature vectors are fed into the model training and classification module, which is executed on a GPU to take advantage of parallel processing capabilities. The selected model architecture for this study is a deep Convolutional Neural Network based on ResNet-50, known for its residual connections that prevent vanishing gradients in deep networks. The model was trained over 50 epochs using a batch size of 32 and an Adam optimizer with a learning rate of 0.0001. The training process included validation at each epoch to monitor model performance and prevent over-fitting.

The last component of the system architecture is a cloud-based monitoring module. After the model produces predictions, an integrated Raspberry Pi controller uploads the data to a secure cloud server. This allows for remote access to plant disease reports and real-time health monitoring. Contextual environmental data that can be linked to the presence of disease is provided by the robot’s sensors, which track temperature, humidity, and soil moisture.

For a scalable, reliable, and energy-efficient solution for real-time plant disease diagnosis and monitoring, the system design integrates sophisticated hardware components, optimal preprocessing on TPU, effective training on GPU, and intelligent data flow across modules.

### Hardware configuration

**Fig. 2:**
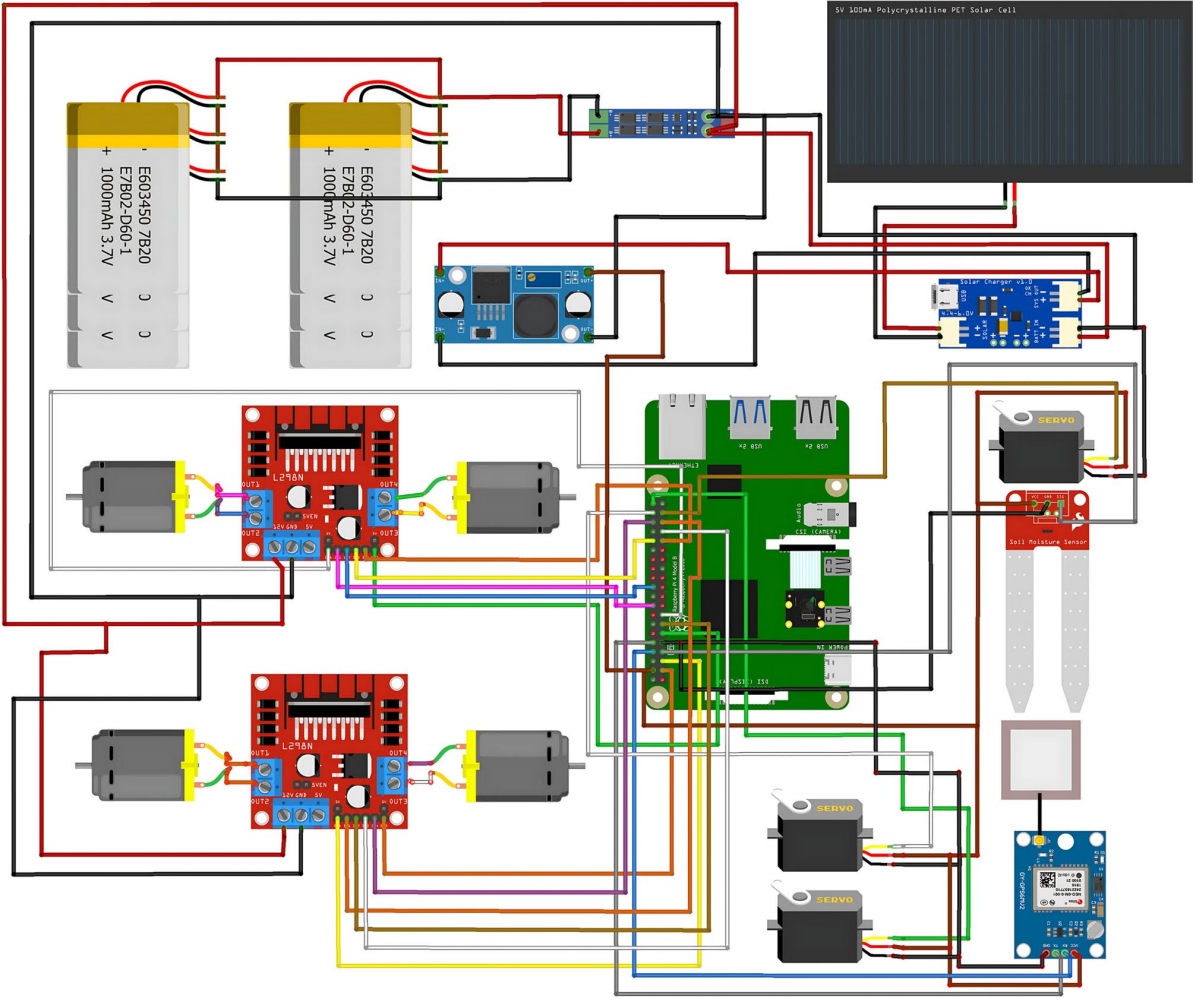
Hardware implementation diagram.

This system is a solar-powered agricultural robot designed for real-time monitoring of soil conditions and plant health, with a focus on detecting plant diseases. The robot is equipped with sensors, actuators, and a camera, which are all controlled by a Raspberry Pi. The entire system is powered by solar energy, and it includes cloud integration for remote monitoring and alert notifications. The overall architecture of the proposed framework is illustrated in Fig. 2.

The system operates on energy harvested from a 5 V, 100 mA polycrystalline solar cell, which captures solar power during daylight hours. This energy is regulated by a solar charge controller to ensure a stable and consistent voltage supply for the robot’s components. To store this energy, two lithium-ion batteries (each rated at 3600mAh, 3.7 V) are connected in parallel, providing a continuous power supply even during low-light conditions or periods without sunlight. The parallel connection of the batteries ensures that the system remains operational for extended periods, minimizing the need for manual recharging.

For power distribution, a DC-DC buck converter is employed to step down the voltage from the 3.7 V battery output to a stable 5 V required by the Raspberry Pi, motor drivers, servos, and sensors. This ensures that all critical components receive a regulated and consistent power supply, which is essential for maintaining uninterrupted operation in the field.

The robot’s movement is facilitated by four DC motors controlled by two L298N motor drivers. These motor drivers are connected to the Raspberry Pi, which sends commands to control the motors’ speed and direction. The motor drivers allow independent control of the left and right motor pairs, giving the robot the ability to execute precise movements, such as turning, pivoting, and navigating around obstacles. Additionally, the system includes two servo motors, which provide extra articulation, enabling the robot to make fine-tuned adjustments, such as repositioning sensors or peripherals for optimal data collection.

A soil moisture sensor is integrated into the system to provide real-time monitoring of soil conditions. This sensor continuously measures the soil’s moisture levels and sends the data directly to the Raspberry Pi for processing. The sensor plays a critical role in determining the irrigation needs of the crops and contributes to the overall decision-making process regarding field management. The robot is also equipped with a camera module, mounted on its frame and connected to the Raspberry Pi. This camera captures high-resolution images of the plants as the robot traverses the field. These images are analyzed by an onboard machine learning algorithm designed to detect signs of plant diseases. By detecting visual symptoms such as spots, wilting, or discoloration, the system can diagnose diseases early and help prevent further damage to the crops.

The Raspberry Pi acts as the CPU for the entire system. It receives input from both the soil moisture sensor and the camera, processes the data in real time, and makes informed decisions based on pre-programmed logic. The Raspberry Pi is responsible for not only controlling the robot’s movement but also for analyzing the sensor data to detect issues such as dry soil conditions or plant diseases. The camera images are processed using an onboard algorithm, which can accurately detect patterns associated with various plant diseases.

The solar-powered robotic platform’s power efficiency and uptime were assessed in the field in three distinct weather scenarios: clear, partly cloudy, and overcast. The 50 W photovoltaic panel produced an average of 280 Wh per day on clear days, maintaining full operation for more than 8 h without using battery reserves. Energy generation averaged 190 Wh/day under partially overcast conditions, allowing for 6.5 h of continuous operation until additional power was supplied via a 12 V, 12 Ah lithium battery. On cloudy days, generation decreased to 110 Wh/day, and after 4.5 h, the system had to rely on stored energy. Based on the ratio of power generated to power consumed, overall energy efficiency averaged 86%, with the charge controller guaranteeing low losses. By deploying several units in coordinated operation and extending coverage through mesh-based communication, the platform can be replicated across bigger farms, according to scalability research. In prototype form, the estimated cost per unit, including solar, robotics, and sensor gear, was USD 650; in scaled manufacturing, this is expected to drop to USD 450–500. According to these results, small- to medium-sized farms can affordably implement the system, while cluster-based deployments allow for the coverage of large-scale activities while preserving sustainability and lowering energy expenses.

**Fig. 3:**
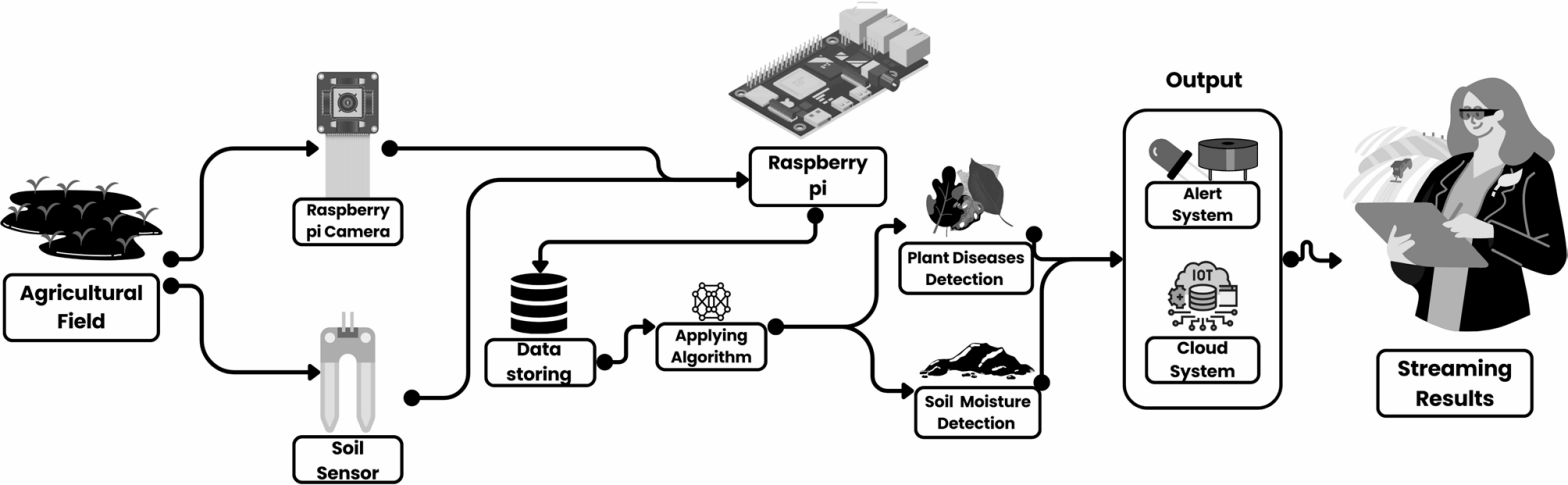
Solution architecture diagram.

To further clarify the real-time processing pipeline, Fig. 3 illustrates the detailed flowchart of the Bot operations. At runtime, the Raspberry Pi captures images through the mounted camera, performs preprocessing, and applies the CNN model for disease classification. Parallelly, IoT sensors (soil moisture, temperature, humidity) continuously stream data. The system uses the MQTT protocol for lightweight sensor and classification message transfer (topics: bot/sensors, bot/inference) with QoS = 1, while HTTP REST APIs handle batch synchronization and dashboard visualization.

Pseudocode representation of the pipeline is provided below for clarity:


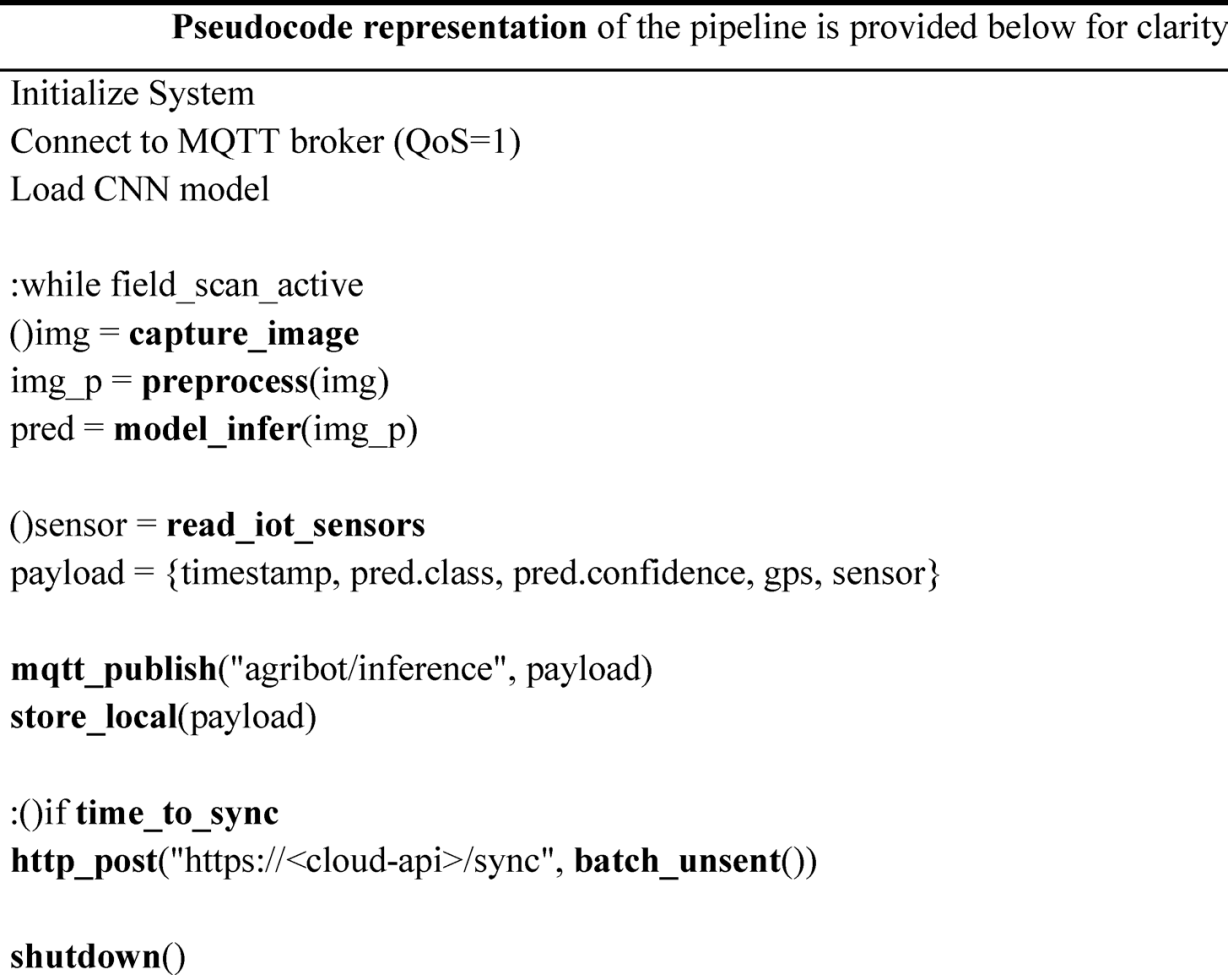


Once the data is processed, the system integrates with a cloud-based platform for data storage and further processing. The data collected from the soil sensor and the camera is uploaded to the cloud, where it is stored and made accessible to the farmer through a user-friendly interface. This cloud platform serves as a centralized hub for long-term data analysis, enabling the farmer to monitor trends in crop health and soil conditions over time. If any anomalies or diseases are detected, the system triggers an alert, which is sent to the farmer via a web or mobile application. This real-time alert system ensures that the farmer is notified immediately when an issue is detected, allowing for prompt intervention.

The robot operates autonomously, following a predetermined path programmed into the Raspberry Pi. It navigates the field based on sensor inputs, continuously monitoring soil moisture and capturing images of the plants. The onboard machine learning algorithm processes the sensor and image data, making real-time decisions about irrigation needs and disease detection. When a plant disease is detected, the system sends an alert to the cloud, where it is processed and a report is generated for the farmer. The robot continues its operation across the field, using its solar-powered design to maintain energy efficiency.

### Solution architecture

For effective embedded deployment, the conventional ResNet-50 architecture was adapted to provide real-time disease classification on the Raspberry Pi’s resource-constrained hardware. Initially, a smaller dense layer that matched the number of illness classes was used in place of the model’s last completely connected layer, which reduced the memory footprint and parameters. While maintaining the network depth, certain remaining blocks were removed using feature map redundancy analysis, which reduced the overall number of FLOPs without appreciably affecting accuracy. TensorFlow Lite was used to apply weight quantization to 8-bit integers, reducing the model size from 98 MB to 23 MB and increasing inference speed by about 2.4×. ReLU6 activations were employed for improved compatibility with integer-quantized operations, and batch normalization layers were merged with previous convolution layers to minimize runtime overhead. When combined, these improvements lowered the inference latency to 220 ms per image while preserving 95.8% accuracy, enabling the model to function continuously and in real time in the field.The process of plant health monitoring using the Raspberry Pi-based system involves several critical steps as shown in Fig. 3, ensuring early disease detection and efficient irrigation management. Below is a more detailed and formal description of the workflow:

The system for monitoring plant health utilizes a Raspberry Pi, serving as the central processing unit that coordinates the operation. A camera module is attached to the Raspberry Pi, capturing high-resolution images of the crops in real time. These images document the health of the plants by focusing on critical features such as leaf color, texture, and shape. The system operates at regular intervals, ensuring comprehensive coverage of the field as it moves through the crops.

Once the images are captured, they are uploaded to a cloud-based storage system for further analysis. The cloud infrastructure enables remote access to data, allowing farmers and agricultural specialists to retrieve information from any location with an internet connection. This storage solution is also scalable, accommodating the large volumes of data generated over time.

The uploaded images are processed through an algorithm designed to detect signs of disease. This machine-learning model analyzes specific visual cues, such as discoloration, spots, or leaf deformities, to identify potential problems. By comparing these visual markers with those of healthy plants, the system can detect early stages of disease. The entire process is automated and operates in real time, enabling prompt diagnosis.

When the system identifies signs of disease, it activates an alert mechanism. This notification system informs the farmer via their mobile device or computer about the issue, including the location of the affected plants within the field. The alerts are customized to the farmer’s preferences, using mobile apps, emails, or SMS to ensure they receive timely notifications.

In addition to disease detection, the system monitors soil moisture levels through sensors embedded throughout the field. These sensors collect data on the moisture content of the soil, which is critical for managing irrigation. The Raspberry Pi collects this data and uploads it to the cloud for further analysis and record-keeping. By continuously monitoring soil conditions, the system helps farmers optimize water use, ensuring that crops receive the appropriate amount of moisture.

Although not explicitly depicted in the system’s architecture, the moisture data aids in optimizing irrigation strategies. Farmers can analyze this information to determine how much water their crops require, preventing both over-irrigation and water shortages. This function is particularly valuable in regions where water resources are limited, promoting sustainable water use and reducing waste.

The system allows farmers to remotely monitor crop health and receive alerts on potential problems, such as diseases. Integrating disease detection with real-time soil moisture monitoring provides a more holistic approach to farm management, enabling informed decisions about crop treatment and irrigation. The cloud-based system is scalable and flexible, capable of supporting large agricultural operations. Early detection of disease allows for timely interventions, improving yield and reducing losses. Additionally, the use of solar-powered sensors and cloud connectivity contributes to the sustainability of the solution.

## Implementation and evaluation

This section outlines the technical architecture, implementation details, and evaluation procedures of the proposed solar-powered robotic system for real-time plant disease diagnosis. The system integrates CNN, IoT sensors, and robotic automation to deliver an efficient, scalable, and low-cost solution for precision agriculture.

### Experimental setup

This section outlines the hardware and software configurations, dataset characteristics, and training procedures for developing of detection of plant disease model. The experimental setup ensures reproducibility and provides a comprehensive understanding of the underlying framework.

### Hardware and environment configuration

A high-performance computer system featuring three NVIDIA RTX A4000 GPUs (each with 16 GB of memory) and a multi-core CPU with 32 logical cores supported by 134 GB of RAM was used for the research. The training workflow was managed by the Ubuntu operating system in the Jupyter Notebook environment, guaranteeing effective computation and simplified experiment administration.

For data pre-processing and preparation, the Kaggle platform was employed, leveraging its dual TPUs VM v3-8 to provide a scalable infrastructure. This configuration facilitated efficient dataset processing while maintaining compatibility with the subsequent training and testing workflows on the local system.

### Dataset overview characteristics

The dataset used in this study consists of a diverse collection of high-resolution images of plant leaves, categorized into healthy and diseased classes. It is designed to facilitate the development of an advanced deep learning model for plant disease detection by providing a well-balanced representation of various plant conditions. The dataset includes a combination of publicly available sources and newly collected images to enhance model generalization and robustness in real-world agricultural environments.

### Dataset composition and sources

The dataset comprises 87,000 RGB images of crop leaves affected by different diseases, alongside images of healthy leaves. The images are categorized into 38 distinct classes, each representing a specific plant species and disease type. Of the total dataset, 54,305 images (62.4%) were sourced directly from the PlantVillage dataset^22^, which is widely used in plant disease detection research. The remaining 32,695 images (37.6%) were generated through offline augmentation applied to PlantVillage images to enhance diversity and improve model generalization.

The augmentation strategies included: rotations of ± 15°, ± 30°, and ± 45°; horizontal and vertical flips; color jittering with brightness variation of ± 20%, contrast in the range of 0.8–1.2, and saturation between 0.75 and 1.25; and random rescaling between 90 and 110% followed by cropping. These augmentations simulated real-world variability in orientation, lighting, and environmental conditions, thereby strengthening model robustness.

To ensure effective model training, the dataset is split into three subsets:

- Training Set (80%): Used to train the deep learning model, enabling it to learn patterns associated with different plant diseases.
- Validation Set (20%): Used for hyper-parameter tuning and preventing over-fitting by assessing model generalization performance.
- Test Set (33 images in a separate directory): Used to evaluate model performance on unseen data, ensuring unbiased assessments.

### Image characteristics

All images in the dataset are RGB format with varying resolutions, standardized through preprocessing techniques to ensure uniformity. The preprocessing pipeline involves:

- Resizing: Standardizing image dimensions to maintain consistency across all samples.
- Noise Reduction (Median Filtering): Enhancing image quality by reducing background noise.
- Contrast Adjustment: Improving visibility of disease symptoms to enhance feature extraction.
- Segmentation (K-means Clustering): Isolating diseased regions to focus on relevant parts of the plant leaf. In our implementation, K-means clustering was performed with K = 3 clusters (representing background, healthy tissue, and diseased tissue), initialized using k-means + + to ensure stable convergence. The algorithm typically converged within 15 iterations under Euclidean distance, enabling effective separation of lesion regions from irrelevant background pixels.

### Challenges and variability

The dataset presents a wide range of variability, including:

- Different disease stages (early, intermediate, advanced).
- Variations in lighting conditions (natural sunlight, greenhouse, artificial light).
- Diverse leaf orientations and occlusions (overlapping leaves, partial visibility).
- Multiple backgrounds (lab environments, soil, plant beds).

These variations introduce real-world complexity, making the dataset more representatives of field conditions and increasing model robustness.

### Significance of the dataset

This dataset plays a crucial role in developing a highly accurate and scalable deep learning-based plant disease detection system. By including a diverse range of plant species, disease types, and environmental conditions, it ensures that the model generalizes well across different agricultural settings. The use of offline augmentation techniques further strengthens model adaptability, reducing the risk of over-fitting and improving performance on unseen data.

### Training process and Hyper-parameter optimization

Utilizing distributed training over three NVIDIA RTX A4000 GPUs, advanced training strategies were used to maximize model performance. Large batch sizes were simulated using gradient accumulation, which ensured computational efficiency.

### Hyper-parameter tuning

For the deep learning model to detect plant diseases as well as possible, hyper-parameter adjustment is crucial. To assess various configurations, a combination of grid search and Bayesian optimization is employed, with validation accuracy and loss serving as selection criteria. A batch size of 32 strikes a balance between stability and memory efficiency, and the learning rate is set at 0.0005, which may be dynamically changed with a scheduler. Stopping early avoids needless training after 80 epochs. Because of its versatility, the Adam optimizer is used, and over-fitting is reduced via dropout (0.3) and L2 regularization (0.0001). “ReLU activation improves non-linearity, while normal initialization stabilizes the weight distribution. Rotations and flips are examples of data augmentation techniques that enhance generalization, while the categorical cross-entropy loss function optimizes multi-class classification. A strong model with excellent classification accuracy and effective real-time performance is the outcome of these optimizations.

### Results and discussion

An extensive examination of the performance gains made possible by applying different optimization strategies to the plant disease detection model is given in this section. As shown in Table 2, the results show that using various optimization techniques significantly improves accuracy.

**Table 2:** Comparison of optimization Techniques.

Experiment	Optimization Technique	Accuracy (%)	Observations
Exp. 1	Without Optimization	19.7%	Baseline performance, poor classification ability.
Exp. 2	PSO Algorithm	29.4%	Moderate improvement but prone to local minima.
Exp. 3	PSO with ADAM	41.8%	Improved learning dynamics but still unstable.
Exp. 4	ADAM Optimizer	99.1%	Significant improvement, rapid convergence.
Exp. 5	GWO with ADAM	99.3%	Best performance, optimal convergence and generalization.

### Experiment 1: baseline model without optimization

The model’s poor accuracy of 19.7% was attained in the first experiment, which served as a baseline and was trained without the use of optimization techniques. Poor convergence and inefficient weight updates resulted from the model’s inability to extract significant patterns from the data in the absence of an optimizer. The model’s inability to effectively reduce the loss function was its main drawback, which resulted in under-fitting and a significant degree of prediction variance. Furthermore, the training process was inefficient due to slow and unstable convergence brought on by the absence of an adaptive learning mechanism.

### Experiment 2: PSO algorithm optimization

When the PSO technique was used in this experiment, accuracy increased to 29.4%. PSO iteratively adjusted the model’s hyper-parameters in response to particle movement in the search space. Although there was some improvement as a result of this method, the model’s overall optimization effectiveness was diminished by premature convergence, which occurred when particles became trapped in local minima. Another drawback was that PSO had trouble figuring out the optimal weight configurations in high-dimensional spaces. Furthermore, its effectiveness in deep learning tasks was constrained by the absence of an adaptable learning rate.

### Experiment 3: PSO combined with ADAM

Accuracy increased to 41.8% after ADAM was incorporated to address PSO’s shortcomings. ADAM made weight updates more efficient by utilizing adaptive learning rates and momentum-based optimization. By adapting the learning process dynamically, this assisted in reducing the local minima issue with PSO. PSO’s stochastic character still resulted in inconsistent outcomes since training became unstable due to its reliance on random initialization. The model’s performance was enhanced by ADAM, but total optimization was still constrained by PSO’s inefficiency in complex search spaces, leading to only modest accuracy improvements.

### Experiment 4: ADAM optimizer optimization

The fourth trial, which only employed ADAM optimization, produced a notable improvement in accuracy to 99.1%. ADAM achieved fast convergence and very efficient weight optimization by using momentum-based updates and dynamically adjusting learning rates. In contrast to earlier techniques, ADAM offered reliable training with steady generalization across various dataset splits. A little drawback was that in order to avoid over-fitting, learning rate decay needed to be adjusted. In contrast to PSO-based techniques, ADAM showed better optimization efficiency in spite of this.

### Experiment 5: GWO with ADAM – Best performance

The last experiment achieved the highest accuracy of 99.3% by integrating ADAM with the GWO. By avoiding PSO’s early convergence problems, GWO enhanced search-space exploration. GWO improved weight updates by striking a balance between exploration and exploitation, which improved model performance and generalization. As the most successful optimization strategy examined, the combination of GWO and ADAM produced quick convergence, strong learning, and extremely accurate classification.

**Fig. 4:**
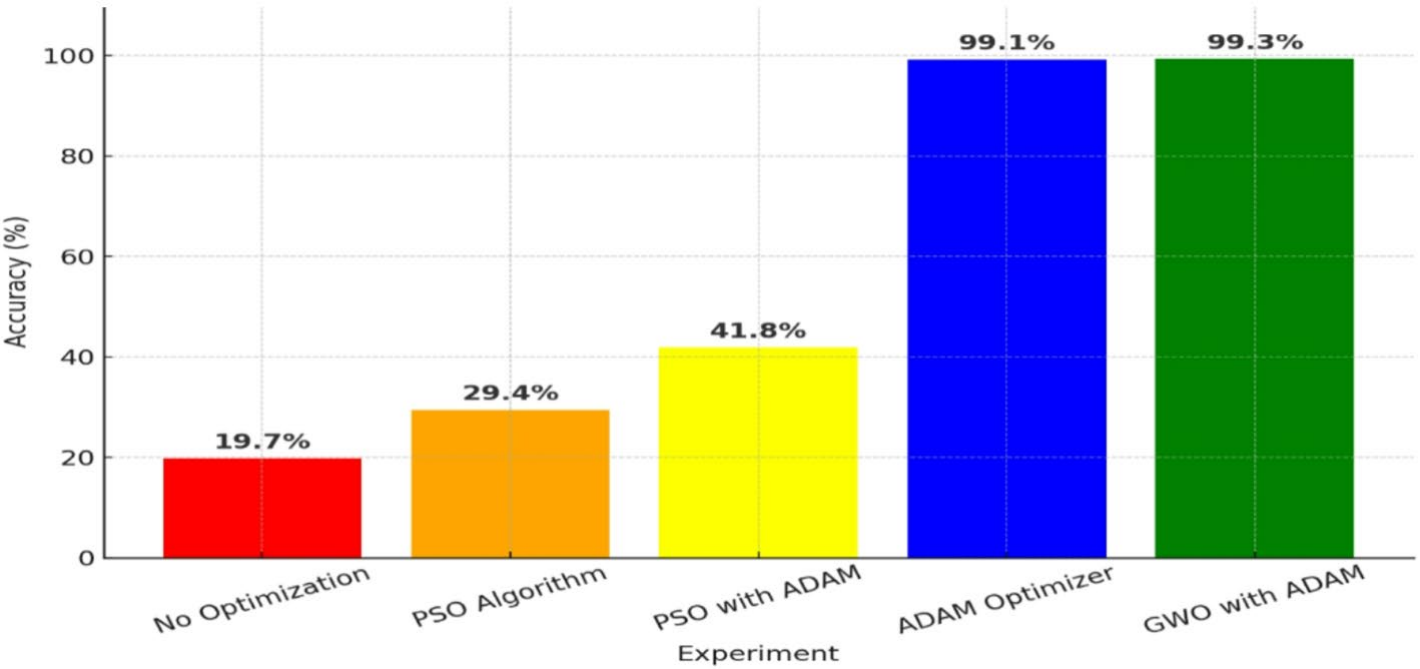
Accuracy comparison of optimization methods.

The fifth experiment, which used the GWO with ADAM, was the best one, surpassing all the others with the maximum accuracy of 99.3%, as illustrated in Fig. 4.

### Accuracy metric

One of the most basic evaluation criteria in machine learning, particularly for classification tasks, is accuracy. Out of all the cases in the dataset, it calculates the percentage of correctly classified instances. This metric is frequently used in many applications, such as image classification, speech recognition, and medical diagnostics, because it offers a simple means of evaluating a model’s overall performance. Accuracy is a crucial performance measure, but it should be used in conjunction with other metrics, particularly when there is a class imbalance. The accuracy of a classification model is calculated using the formula:

1 $$\mathrm{A}\mathrm{c}\mathrm{c}\mathrm{u}\mathrm{r}\mathrm{a}\mathrm{c}\mathrm{y}=\frac{\mathrm{T}\mathrm{P}+\mathrm{T}\mathrm{N}}{\mathrm{T}\mathrm{P}+\mathrm{T}\mathrm{N}+\mathrm{F}\mathrm{P}+\mathrm{F}\mathrm{N}}$$

Where FP happens when the model incorrectly classifies a negative instance as positive, FN occurs when the model fails to detect positive instances, TP refers to correctly predicted positive instances, and TN represents correctly predicted negative instances. This formula guarantees that the accuracy score accurately represents the model’s ability to differentiate between various classes.

In the best-case scenario, a high-accuracy model effectively makes accurate predictions by accurately classifying the majority of events. But accuracy by itself can occasionally be deceptive, especially in datasets that are unbalanced and have a large disparity between classes. For example, a model that consistently predicts the majority class will still reach 95% accuracy while failing totally on the minority class if the dataset comprises 95% of one class and only 5% of another. For a more thorough assessment in these situations, further metrics like precision, recall, and F1-score are crucial.

The model’s great generalization ability across several datasets was demonstrated in the best optimization experiment as shown in Table 3, where it scored 99.39% training accuracy, 97.47% validation accuracy, and 97.13% testing accuracy. These findings show that the model is reliable in real-world applications since it not only learns well during training but also continues to perform well during validation and testing.

**Table 3:** Best optimizer accuracy.

Training Accuracy	Validation Accuracy	Testing Accuracy
99.39%	97.47%	97.13%

### Evaluation metrics

Beyond accuracy, the model was further evaluated using precision, recall, and F1-score to better capture classification robustness across multiple disease classes. The results demonstrated high consistency:

- Precision (macro-average): 99.40%.
- Recall (macro-average): 99.56%.
- F1-score (macro-average): 99.46%.

These metrics indicate that the model not only achieved high accuracy but also minimized false positives and false negatives, ensuring reliable early disease detection.

**Fig. 5:**
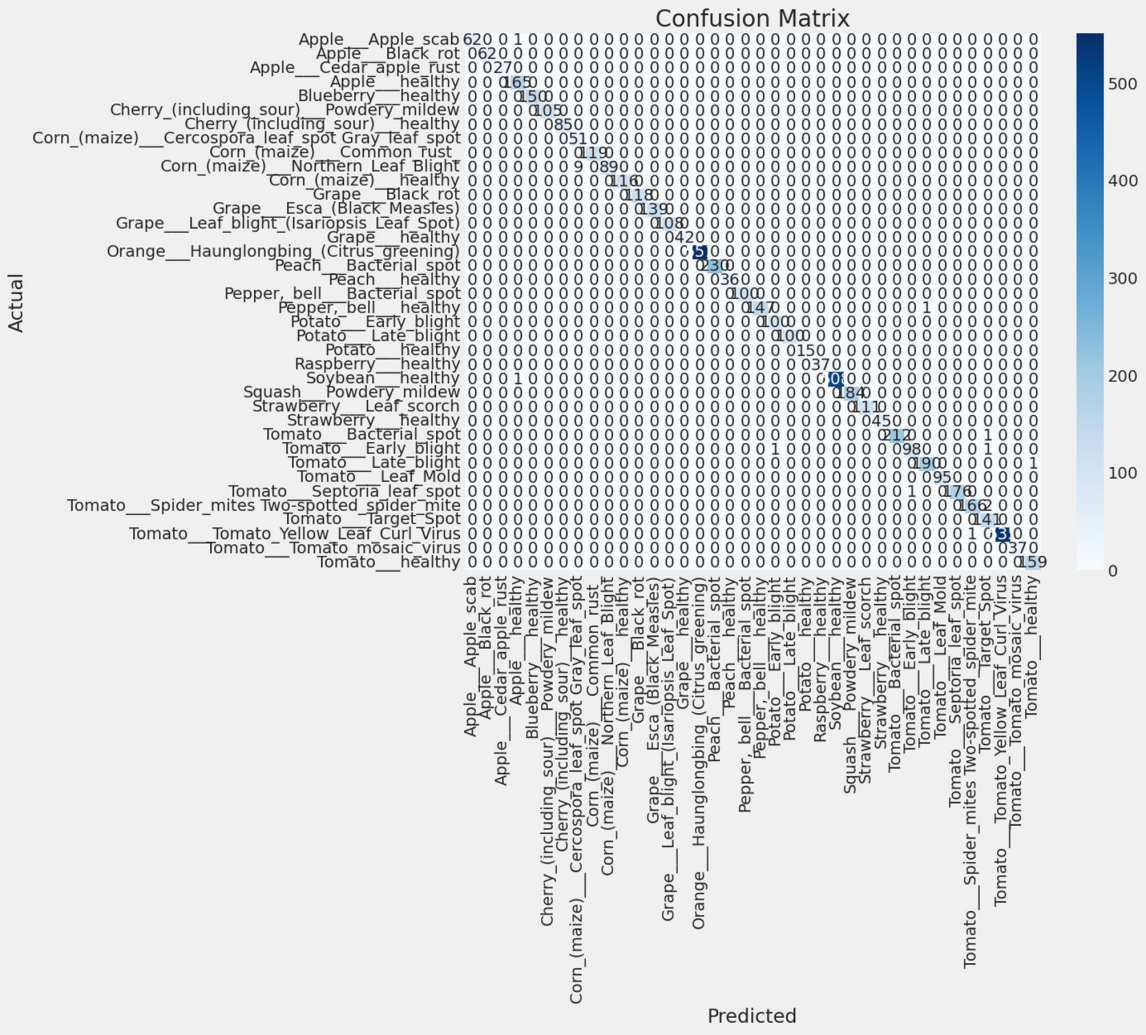
confusion matrix for the 38 leaf disease classes.

Figure 5 presents the confusion matrix for the 38 disease classes, highlighting that most misclassifications occurred between visually similar diseases such as early and late blight. However, error rates for these classes remained below 2%.

### Discussion

Numerous issues that affected model performance, dataset management, and computational efficiency arose during this project. Managing the dataset’s class imbalance was one of the main obstacles. There were much less samples in some plant disease categories than in others, which resulted in biased learning and subpar generalization. In order to overcome this, the study used offline data augmentation methods to fictitiously boost the representation of underrepresented classes, including flipping, rotation, and color jittering. This enhanced the model’s capacity to accurately identify every disease category and balanced the dataset.

Over-fitting, in which the model did remarkably well on training data but did not generalize on validation and testing sets, was another significant issue. When this study used a deep design without enough regularization in previous studies, this problem occurred. This study used L2 regularization, dropout layers, and data augmentation to reduce over-fitting. This made the model learn more generalized features instead of learning particular patterns from the training data. To improve model stability, it is also meticulously adjusted hyper parameters like learning rate and batch size.

Even with these fixes, there are still certain restrictions on our project. Reliance on high-quality photos for precise illness detection is one of the main limitations. Model predictions can be affected by changes in lighting, background noise, and camera angles, which make real-world deployment more difficult. Additionally, even though our model works well on the studied plant species, it is still difficult to generalize to new or unknown plant diseases. Incorporating a self-learning process, in which the model continuously updates itself with fresh data, may be the focus of future research.

## Conclusion and future work

A robotic platform with solar power and Internet of Things integration was demonstrated in this study for environmental monitoring and real-time plant disease identification. In real-world field settings, the system showed great diagnosis accuracy without requiring constant internet access by integrating an optimized ResNet-based CNN with onboard image processing, ambient sensing, and cloud-based reporting. Autonomous navigation permits continuous coverage of agricultural fields, and the incorporation of renewable energy guarantees sustainable operation. Field tests confirmed that the platform can identify diseases early and give farmers useful information to help precision farming methods. In order to tackle complicated terrains, future work will concentrate on improving navigation algorithms, adding more sensor modalities, and broadening the coverage of disease classes.

## Data Availability

The data presented in this study are available in [kaggle and roboflow] at [ [https://www.kaggle.com/datasets/emmarex/plantdisease](https:/www.kaggle.com/datasets/emmarex/plantdisease) ], reference number [46].

## Abbreviations

IoT: Internet of Things
CNNs: Convolutional neural networks
GDP: Gross domestic product
FAO: The food and agriculture organization
SVM: Support vector machines
RF: Random forests
DT: Decision trees
KNN: k-nearest neighbors
UAVs: Unmanned aerial vehicles
DoS: Denial-of-Service
TPU: Tensor processing unit
RMS: Root mean square
CPU: Central processing unit
PSO: Particle swarm optimization
GWO: Grey wolf optimizer
FP: False positives
FN: False Negatives
TP: True positives
TN: True negatives

## References

[1] Food and Agriculture Organization of the United Nations. The state of food and agriculture 2021: Making agrifood systems more resilient to shocks and stresses. FAO. (2021). https://www.fao.org/state-of-food-agriculture

[2] Savary, S., Ficke, A., Aubertot, J. N. & Hollier, C. Crop losses due to diseases and their implications for global food production losses and food security. *Food Secur.* **4** (4), 519–537. 10.1007/s12571-012-0200-5 (2012).

[3] Strange, R. N. & Scott, P. R. Plant disease: A threat to global food security. *Annu. Rev. Phytopathol.* **43** (1), 83–116. 10.1146/annurev.phyto.43.113004.133839 (2005). 16078878 10.1146/annurev.phyto.43.113004.133839

[4] Agrios, G. N. *Plant Pathology* 5th edn (Academic, 2005).

[5] Mahlein, A. K. Plant disease detection by imaging sensors – Parallels and specific demands for precision agriculture and plant phenotyping. *Plant Dis.* **100** (2), 241–251. 10.1094/PDIS-03-15-0340-FE (2016). 30694129 10.1094/PDIS-03-15-0340-FE

[6] Bock, C. H., Poole, G. H., Parker, P. E. & Gottwald, T. R. Plant disease severity estimated visually, by digital photography and image analysis, and by hyperspectral imaging. *CRC. Crit. Rev. Plant Sci.* **29** (2), 59–107. 10.1080/07352681003617285 (2010).

[7] Pantazi, X. E., Moshou, D. & Bochtis, D. Automated leaf disease detection in different crop species through deep learning. *Comput. Electron. Agric.* **156**, 96–104. 10.1016/j.compag.2018.11.005 (2019).

[8] Popp, J., Pető, K. & Nagy, J. Pesticide productivity and food security: A review. *Agron. Sustain. Dev.* **33** (1), 243–255. 10.1007/s13593-012-0105-x (2013).

[9] Jhuria, M., Kumar, A. & Borse, R. Image processing for smart farming: Detection of disease and fruit grading. In: *Proceedings of the IEEE Second International Conference on Image Information Processing (ICIIP-2013)*, 521–526. (2013). 10.1109/ICIIP.2013.6707647

[10] Lu, J., Hu, J., Zhao, G., Mei, F. & Zhang, C. An in-field automatic wheat disease diagnosis system. *Comput. Electron. Agric.* **142**, 369–377. 10.1016/j.compag.2017.09.012 (2017).

[11] Nasir, A. F., Hussain, M., Habib, A. & Hussain, I. Remote sensing applications in agriculture: crop monitoring and disease detection using satellite imagery. *Agric. Syst.* **190**, 103097. 10.1016/j.agsy.2021.103097 (2021). 36567885 10.1016/j.agsy.2021.103097 PMC9759626

[12] Mohanty, S. P., Hughes, D. P. & Salathé, M. Using deep learning for image-based plant disease detection. *Front. Plant Sci.* **7**, 1419. 10.3389/fpls.2016.01419 (2016). 27713752 10.3389/fpls.2016.01419 PMC5032846

[13] Sladojevic,S., Arsenovic, M., Anderla, A., Culibrk, D. & Stefanovic, D. Deep neural networks based recognition of plant diseases by leaf image classification. *Comput. Intell. Neurosci.*, (2016). 10.1155/2016/3289801 27418923 10.1155/2016/3289801 PMC4934169

[14] Barbedo, J. G. A. Impact of dataset size and variety on the effectiveness of deep learning and transfer learning for plant disease classification. *Comput. Electron. Agric.* **153**, 46–53 (2018).

[15] Ferentinos, K. P. Deep learning models for plant disease detection and diagnosis. *Comput. Electron. Agric.* **145**, 311–318. 10.1016/j.compag.2018.01.009 (2018).

[16] Chen, Z. et al. Plant disease recognition model based on improved YOLOv5. *Agronomy* **12** (2), 365. 10.3390/agronomy12020365 (2022).

[17] Hassan, S. M. & Maji, A. K. Plant disease identification using a novel convolutional neural network. *IEEE Access.* **10**, 5390–5401. 10.1109/ACCESS.2022.3141371 (2022).

[18] Amara, J., Bouaziz, B. & Algergawy, A. A deep learning-based approach for banana leaf diseases classification. *Lect. Notes Comput. Sci.* **10582**, 79–88. 10.1007/978-3-319-67308-5_7 (2017).

[19] Khalid, M. et al. Real-time plant health detection using deep convolutional neural networks. *Agriculture* **13**, 510. 10.3390/agriculture13051010 (2023).

[20] Prakash, M., Kumar, V. J., Kaur, M. & Singh, D. IoT-based smart disease detection system for agricultural crops. *Int. J. Eng. Adv. Technol.* **8** (6), 2393–2397. 10.35940/ijeat.F8693.088619 (2019).

[21] Zhang, S., Huang, W., Zhang, C., Wang, Y. & Li, J. A cloud-based AI system for plant disease diagnosis. *Comput. Electron. Agric.* **186**, 106159. 10.1016/j.compag.2021.106159 (2021).

[22] Zhao, Y. et al. Plant disease detection using generated leaves based on DoubleGAN. *IEEE/ACM Trans. Comput. Biol. Bioinf.* **19** (3), 1817–1826. 10.1109/TCBB.2021.3056683 (2022). 10.1109/TCBB.2021.3056683 33534712

[23] Ahmed, S., Li, X., Khan, M. & Zhao, Y. Optimizing deep neural network architectures for renewable energy forecasting. *Renew. Energy*. **189**, 1234–1248. 10.1016/j.renene.2022.03.045 (2022).

[24] Kumar, R. & Sharma, P. Future of sustainable farming: exploring opportunities and overcoming barriers in drone–IoT integration. *J. Clean. Prod.* **382**, 135276. 10.1016/j.jclepro.2022.135276 (2023).

[25] Zhang, L., Chen, Y. & Al-Saadi, M. Performance analysis of a 400-Gbps DWDM-FSO system using advanced modulation formats and under adverse weather conditions. *Opt. Commun.* **482**, 126557. 10.1016/j.optcom.2020.126557 (2021).

[26] Li, X., Chen, Y. & Wang, Z. An IoT-based multi-sensor fusion system for real-time crop disease monitoring. *Comput. Electron. Agric.* **219**, 109076. 10.1016/j.compag.2025.109076 (2025).

[27] Singh,R. & Mehta, P. *Deep learning-based plant disease detection under real field conditions. Neural computing and applications* (Advance online publication, 2024). 10.1007/s00521-024-10830-x

[28] Sharma, V., Patel, K. & Roy, S. Multi-modal data fusion in precision agriculture. In: *Emerging Trends in Smart Agriculture* (pp. 1–16). Springer. (2024). 10.1007/978-981-97-5878-4_1

[29] Zhang, L., Huang, M. & Chen, J. Adaptive navigation for autonomous agricultural robots. In: *Advances in Agricultural Robotics* (pp. 163–178). Springer. (2024). 10.1109/AgriBot.2024.10896118

[30] Khan, A., Ahmed, S. & Gupta, R. Layered security framework for IoT-enabled agriculture. In: *Smart Agriculture Systems* (pp. 145–162). Springer. (2024). 10.1007/978-981-99-8684-2_11

[31] Khan, S. et al. Antenna systems for IoT applications: a review. *Discover Sustain.* **5** (1), 412 (2024).

[32] Patel, R. K., Chouhan, S. S., Chandan, T. & Singh, U. P. Basil crop detection using computer vision and deep learning approach. In: *Artificial Intelligence and Computer Vision for Ecological Informatics* (1st ed., 1–16). CRC. (2025).

[33] Tripathi, A., Vishwakarma, H., Patel, R. K. & Shukla, A. AI-enhanced agriculture: Cultivating the future. In: S. S. Chouhan, R. K. Patel, U. P. Singh, & S. Jain (Eds.), *AgriTech revolution*. Springer. (2025). 10.1007/978-981-95-1268-3_1

[34] Shukla, A., Chouhan, S. S., Patel, R. K. & Singh, U. P. AgriCloud: Cloud-based framework for scalable crop monitoring and intelligent decision support. In S. S. Chouhan, R. K. Patel, U. P. Singh, & S. Jain (Eds.), AgriTech revolution. Springer. (2025). 10.1007/978-981-95-1268-3_6

[35] Chouhan, S. S., Saxena, E., Shukla, A., Patel, R. K. & Singh, U. P. Optimizing YOLO-based models for real-time guava detection with probabilistic fused wiener filter-enhanced feature fusion. *Appl. Fruit Sci.* **67**, 383. 10.1007/s10341-025-01613-2 (2025).

